# Supplementary material for: Prion protein promotes copper toxicity in Wilson disease
Source: Nat Commun. 2025 Feb 8;16:1468. doi: 10.1038/s41467-025-56740-x (PMC11807206; doi:10.1038/s41467-025-56740-x)
Supplement: Supplementary file 1 — Supplementary information [file 41467_2025_56740_MOESM1_ESM.pdf]

SUPPLEMENTARY INFORMATION

Prion protein promotes copper toxicity in Wilson disease

Raffaella Petruzzelli[1,2#], Federico Catalano[1,3#], Roberta Crispino[1], Elena V. Polishchuk[1], Mariantonietta Elia[1], Antonio Masone[4], Giada Lavigna[4], Anna Grasso[4], Maria Battipaglia[1], Lucia Sepe[1], Banu Akdogan[5], Quirin Reinold[6], Eugenio Del Prete[1], Diego Carrella[1], Annalaura Torella[1,7], Vincenzo Nigro[1,7], Enrico Caruso[8], Nicole Innocenti[9], Emiliano Biasini[9], Ludmila V. Puchkova[10,11], Alessia Indrieri[1,12], Ekaterina Y. Ilyechova[10, 11], Pasquale Piccolo[1], Hans Zischka[5,6], Roberto Chiesa[4]* and Roman S. Polishchuk[1]*

[1] Telethon Institute of Genetics and Medicine (TIGEM), Pozzuoli, Italy; [2] Scuola Superiore Meridionale (SSM, School of Advanced Studies), Genomics and Experimental Medicine program; [3] Institute of Biosciences and BioResources, National Research Council, Naples, Italy; [4] Laboratory of Prion Neurobiology, Department of Neuroscience, Istituto di Ricerche Farmacologiche Mario Negri IRCCS, Milan, Italy; [5] Institute of Molecular Toxicology and Pharmacology, Helmholtz Center Munich, German Research Center for Environmental Health, Neuherberg, Germany; [6] Institute of Toxicology and Environmental Hygiene, Technical University Munich, School of Medicine and Health, Munich, Germany; [7] Department of Precision Medicine, University of Campania "Luigi Vanvitelli," Naples, Italy; [8] Department of Biotechnology and Life Sciences, University of Insubria, Varese, Italy; [9] Department of Cellular, Computational and Integrative Biology, University of Trento, Povo, TN, Italy; [10] Department of Molecular Genetics, Research Institute of Experimental Medicine, St. Petersburg, Russia; [11] ITMO University, St. Petersburg, Russia; [12] Institute for Genetic and Biomedical Research (IRGB), National Research Council (CNR), Milan, Italy.

[#] Contributed equally to this study; * Co-corresponding authors: roberto.chiesa@marionegri.it and polish@tigem.it

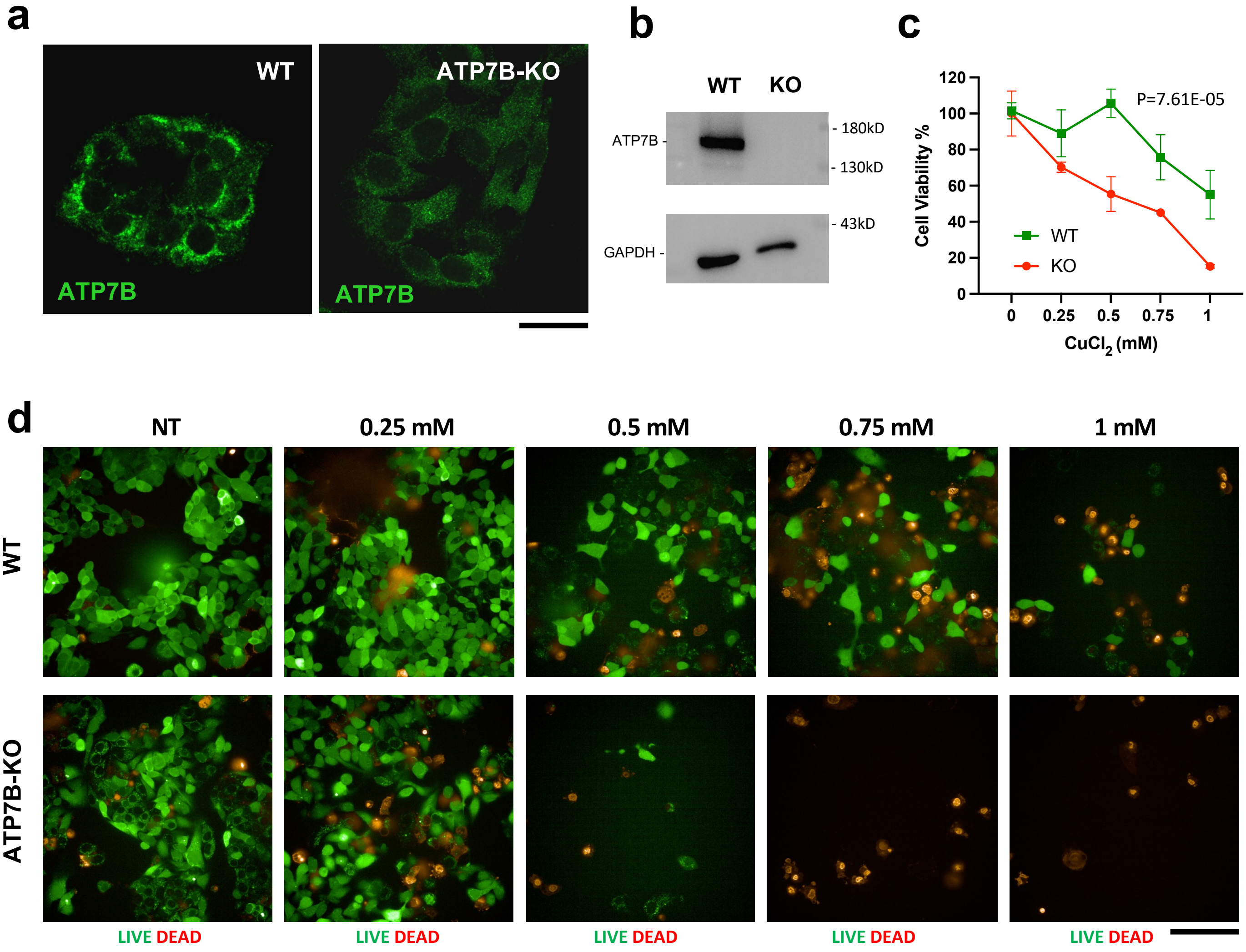

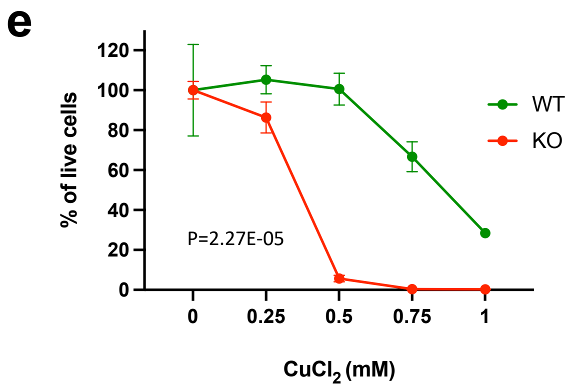

Sup. Fig. 1. Hepatic ATP7B-KO cells exhibit reduced resistance to copper toxicity.

a, b. WT and ATP7B-KO HepG2 cells were prepared for either immunofluorescence (a) or Western blot (b) analysis with AP7B-specific antibody. Both approaches show lack of ATP7B expression in ATP7B-KO cells. **c-e.** WT and ATP7B-KO cells were incubated with $CuCl_2$ at indicated concentrations for 24h and their viability was evaluated by either MTT (c) or live/dead (d, e) assays. The micrographs in panel D demonstrate the lower resistance of ATP7B-KO cells to increasing copper concentrations. Panels c and e show dose response curves corresponding to MTT and Live/Dead assays respectively. Both graphs show higher toxicity of copper in ATP7B-KO cells (two way ANOVA; n=3 experiments). Scale bars: 38 µm (a); 150µm (d).

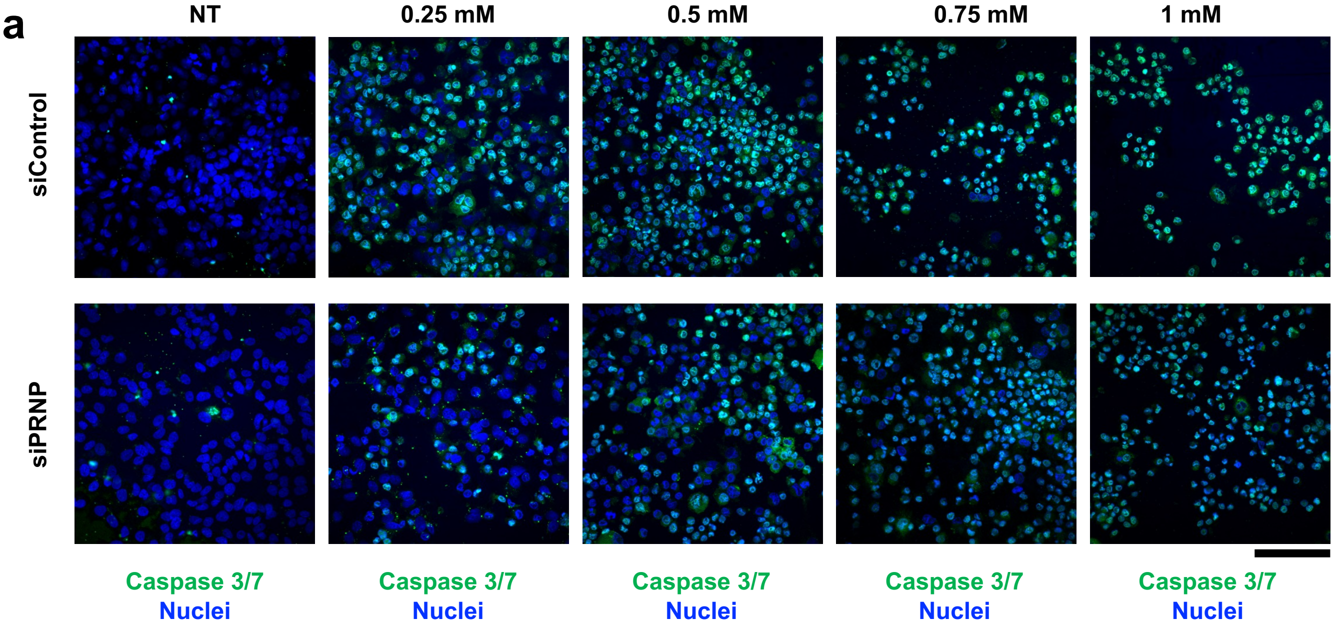

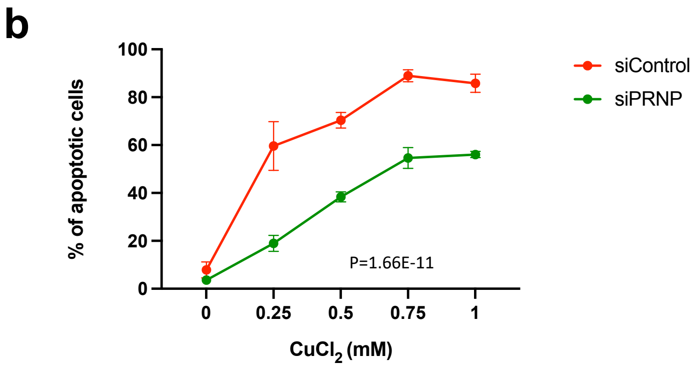

Sup. Fig. 2. PRNP silencing reduces Cu-mediated apoptosis in ATP7B-KO HepG2 cells.

a, b. ATP7B-KO cells were incubated with control (siControl) or *PRNP*-specific siRNAs and exposed to $CuCl_2$ at different concentrations (as indicated in panels and graph) for 24h. Then the cells were stained with CellEvent fluorescent reagent, which reveals activity of caspases 3 and 7 in nuclei of apoptotic cells. Representative micrograms (a) and their quantification (b) show that PRNP suppression reduces % of apoptotic cells upon exposure to Cu (Mean ± SEM; two way ANOVA; n=4 view fields). Scale bar: 300µM (a).

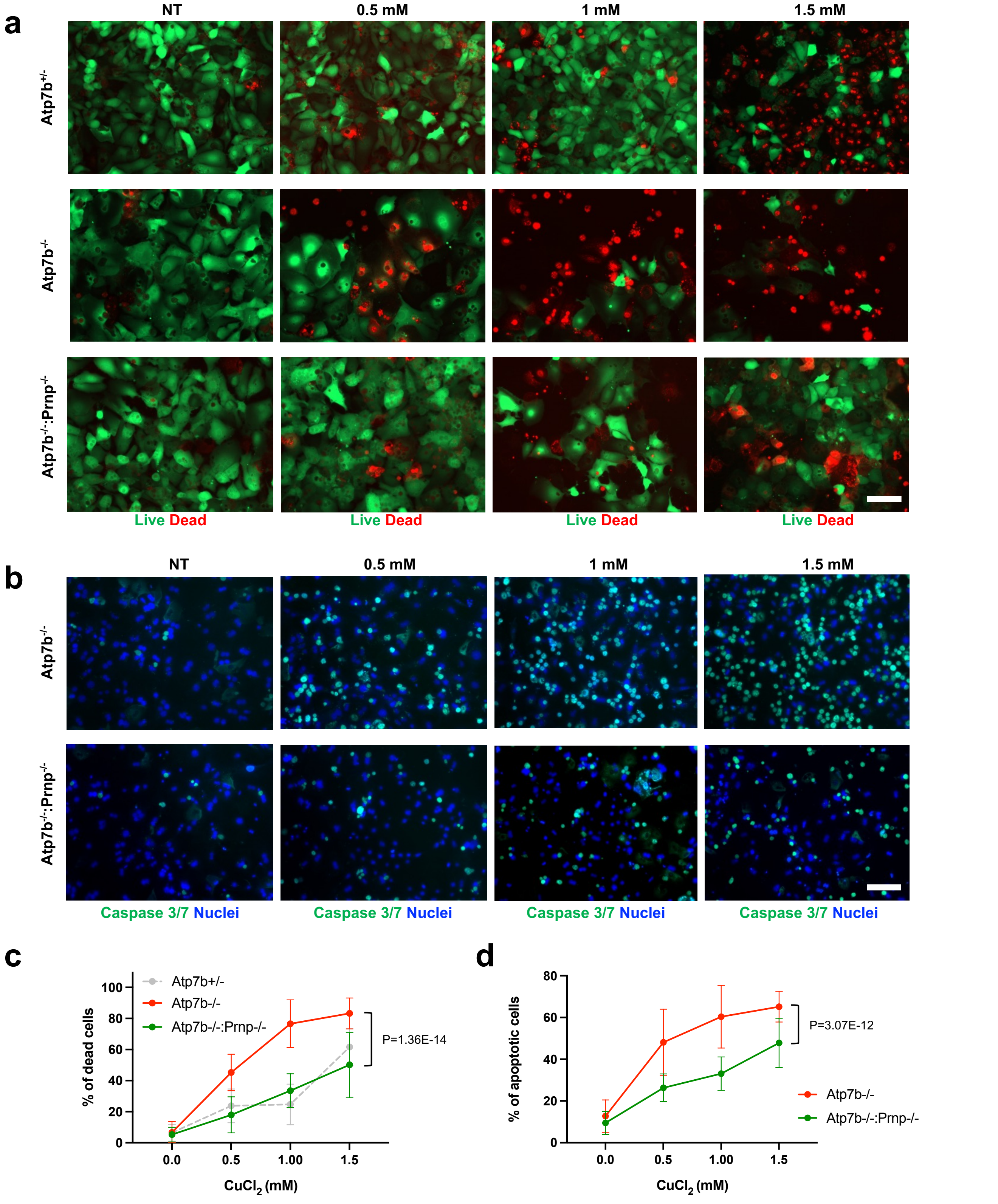

Sup. Fig. 3. Genetic suppression of *Prnp* reduces Cu toxicity in Atp7b-deficient primary hepatocytes.

Primary hepatocytes were isolated from the liver of Atp7b$^{+/-}$ (a), Atp7b$^{-/-}$ (a, b) or Atp7b$^{-/-}$:Prnp$^{-/-}$ (a, b) mice and grown *in vitro*. The hepatocytes were then exposed for 24h to $CuCl_2$ at indicated concentrations and labelled with Live/Dead fluorescent reagent (a) or CellEvent fluorescent kit (b), which reveals activity of caspases 3 and 7 in apoptotic cells. The images show that *Prnp* knockout improves viability of Atp7b$^{-/-}$ hepatocytes (a) and reduces their death (a) and apoptosis (b) upon exposure to Cu. Quantification of live/dead (c) or CellEvent (d) fluorescence shows reduction in Cu-mediated cell death (c) and apoptosis (d) in populations of Atp7b$^{-/-}$:Prnp$^{-/-}$ hepatocytes compared to Atp7b$^{-/-}$ hepatocytes (Mean ± SD; two way ANOVA; n=16 view fields). Scale bar: 150µm (a, b).

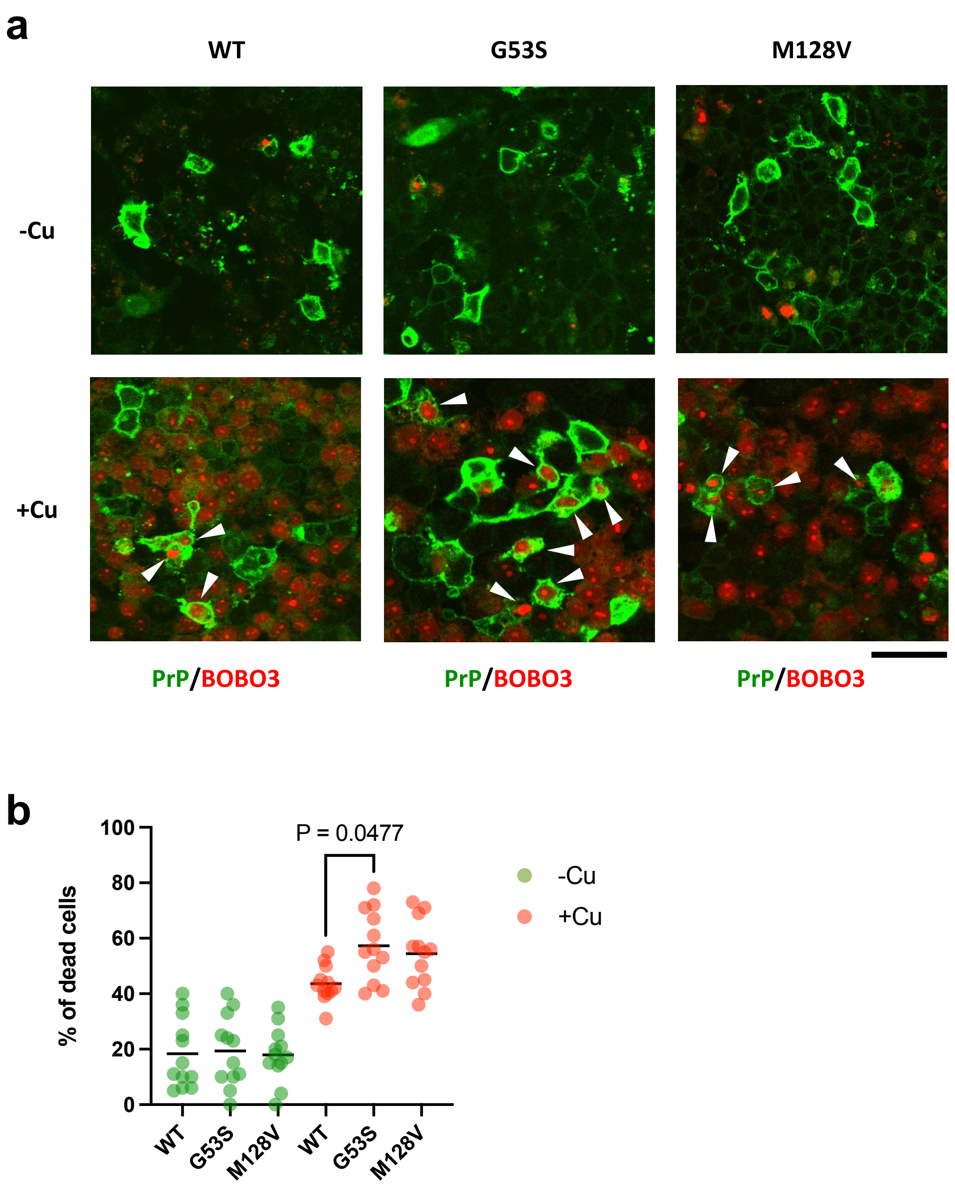

Sup. Fig. 4. Impact of Wilson disease-related PrP variants on Cu toxicity in ATP7B-KO cells.

a, b. ATP7B-KO HepG2 cells with stable PrP knockdown (see methods) were transfected with mouse WT (G53/M128), or G53S, or M128V PrP variants. Then the cells were stained with BOBO3 before (upper row) or after (lower row) CuCl2 treatment (0.5 mM for 24h) to reveal dead cells. Dead cells expressing PrP variants are indicated in panel A by arrowheads. Quantification (b) indicates higher ability of G53S variant to induce Cu-mediated cell death compared to WT protein (Mean; two way ANOVA; n=12 view fields). Scale bar: 27µm (a).

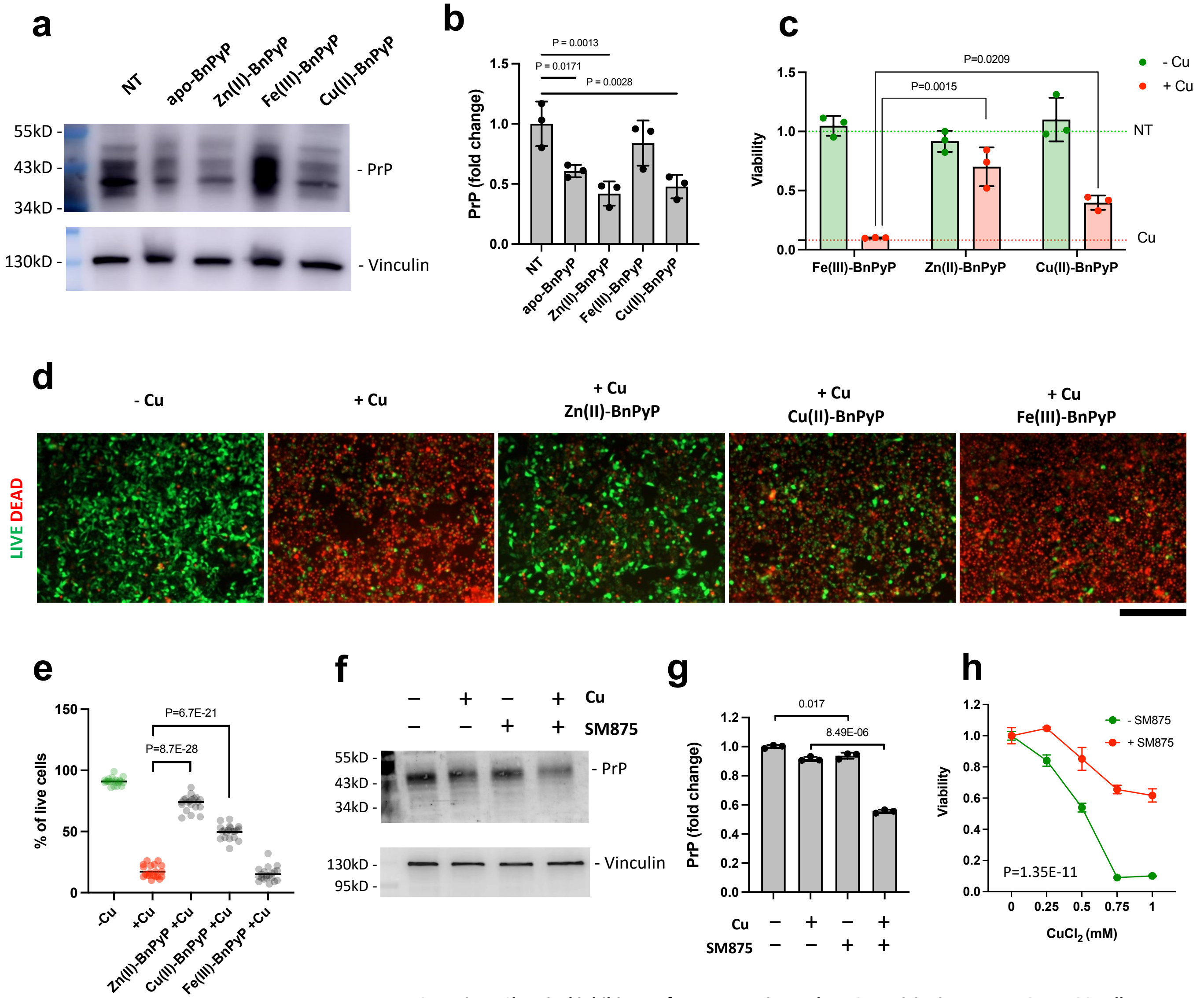

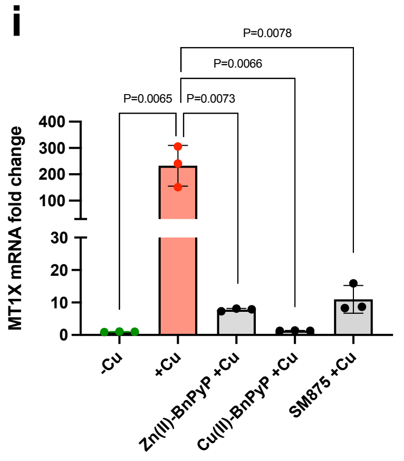

Sup. Fig.5. Chemical inhibitors of PrP expression reduce Cu toxicity in ATP7B-KO HepG2 cells. **a, b.** ATP7B-KO cells were incubated without (NT) or with tetracationic zinc (Zn(II)-BnPyP), copper (Cu(II)-BnPyP), iron (Fe(III)-BnPyP), or empty apo-BnPyP porphyrins (5 µM each) for 24h and cell lysates were analyzed by Western blot analysis using an anti-PrP antibody. Anti-GAPDH was used as a loading control. Quantification shows a significant reduction in PrP levels following treatment with Zn(II)-BnPyP and Cu(II)-BnPyP (Mean ± SD; one-way ANOVA; n=3 experiments). **c.** ATP7B-KO cells treated with 5 µM porphyrins were incubated for 24h with or without 0.5 mM $CuCl_2$ and viability was evaluated using the MTT assay. Values are shown relative to porphyrin-untreated cells without $CuCl_2$ (green dashed line). Red dashed line shows viability levels in cells treated with $CuCl_2$ only (no porphyrins). Zn(II)-BnPyP and Cu(II)-BnPyP significantly reduced Cu toxicity (Mean ± SD; two-way ANOVA; n=3 experiments). **d, e.** ATP7B-KO cells were treated for 24h with 0.5 µM $CuCl_2$ alone or in combination with porphyrins. The cells were then stained with a live/dead fluorescent kit. Representative fluorescent images (d) and their quantification (e) demonstrate that Zn(II)-BnPyP and Cu(II)-BnPyP improve resistance of the cells to Cu (Mean; one-way ANOVA; n=10 view fields). **f, g.** ATP7B-KO cells were incubated with 10 µM SM875, or 0.5 mM $CuCl_2$, or both for 24h. Western blot (f) and quantification (g) show that SM875 reduces PrP levels, especially in Cu-treated cells (Mean ± SEM; one-way ANOVA; n=3 experiments). **h.** The cells were incubated in medium containing 10 µM SM875 (+SM875) or with control medium (-SM875) and then exposed to $CuCl_2$ at different concentrations for 24h. Cell viability was assessed using the MTT assay, which revealed reduction of Cu toxicity in SM875-treated cells (Mean ± SEM; two-way ANOVA; n=3 experiments). **i.** qRT-PCR analysis of MT1X expression in untreated ATP7B-KO cells (-Cu) or cells treated with 0.5 mM $CuCl_2$ alone or in combination with 5 µM Zn(II)-BnPyP, 5 µM Cu(II)-BnPyP, or 10 µM SM875. (Mean ± SD; one-way ANOVA; n=3 experiments). Scale bar: 260 µm (d).

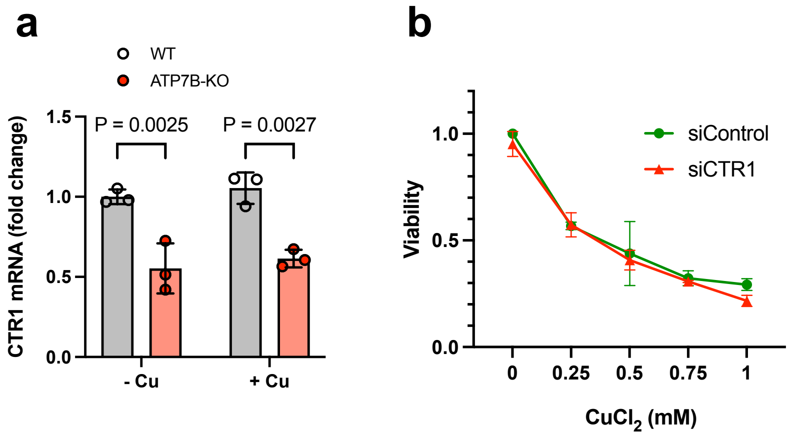

Sup. Fig. 6. ATP7B loss leads to reduced expression of CTR1.

a. *CTR1* expression in WT and ATP7B-KO HepG2 cells. Cells were left intreated or treated with 0.5 mM $CuCl_2$ for 24 h and then processed for qRT-PCR analysis (Mean ± SD, two-way ANOVA; n=3 experiments). **b.** ATP7B-KO cells were incubated with control (siControl) or *CTR1*-specific siRNAs and treated with different concentrations of $CuCl_2$ for 24h. Cell viability was evaluated using the MTT assay, which indicates that *CTR1*-silencing does not improve resistance to Cu (Mean ± SD, two-way ANOVA; n=3 experiments).

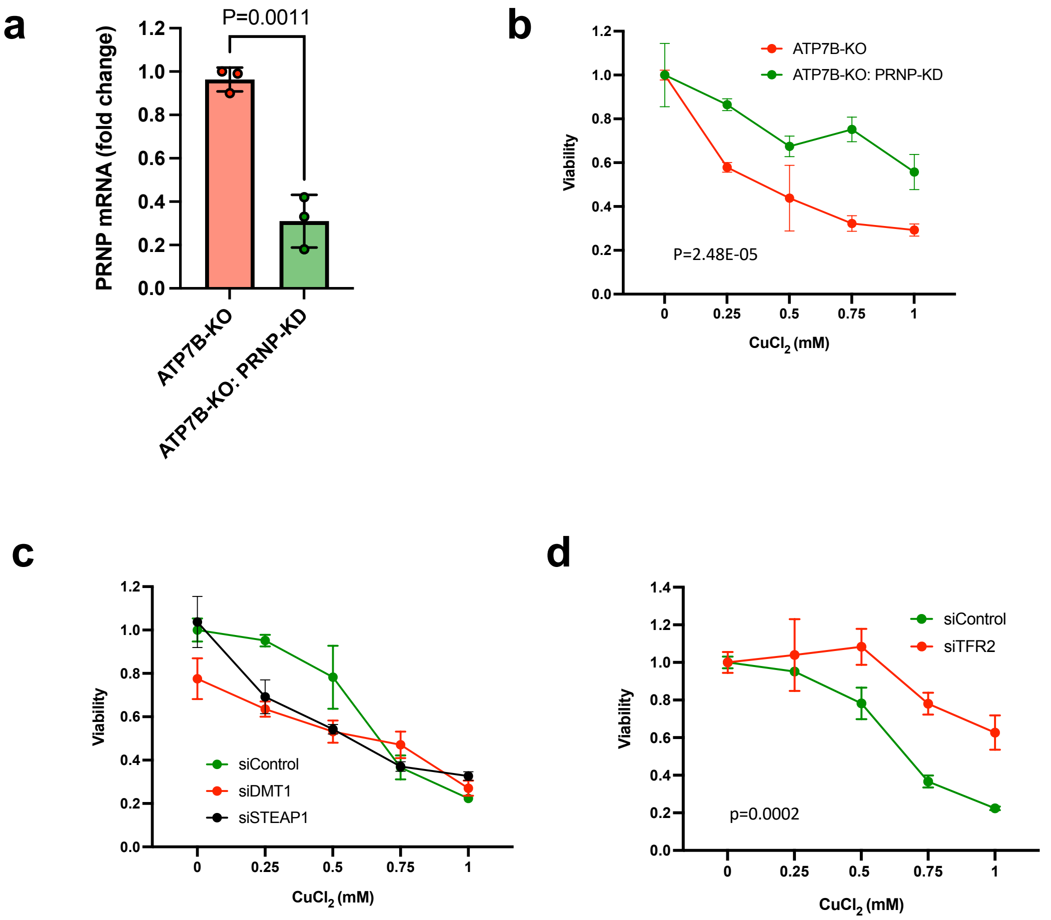

Sup. Fig 7. A combination of PrP suppression with DMT1 or STEAP1 silencing does not have a synergistic impact on Cu resistance in ATP7B-KO cells.

a. qRT-PCR analysis of PRNP expression in the ATP7B-KO line with stable PRNP knockdown (ATP7B-KO:PRNP-KD) and parental ATP7B-KO HepG2 cells (Mean ± SD; t-test; n=3 experiments). **b.** ATP7B-KO:PRNP-KD and ATP7B-KO cells were incubated with $CuCl_2$ at the indicated concentrations for 24h and viability was evaluated using the MTT assay. The graph shows lower toxicity of copper in ATP7B-KO:PRNP-KD line (Mean ± SEM; two-way ANOVA; n=3 experiments). **c.** ATP7B-KO:PRNP-KD cells were incubated with control, DMT1, or STEAP1 siRNAs, exposed to $CuCl_2$ at the indicated concentrations for 24h and analyzed using the MTT assay. Dose response curves indicate that neither DMT1 nor STEAP1 RNAi improves tolerance of the cells to Cu (Mean ± SEM; two-way ANOVA; n=3 experiments). **d.** ATP7B-KO:PRNP-KD cells were treated with control or TFR2 siRNAs, exposed to $CuCl_2$ at the indicated concentrations for 24h and analyzed using the MTT assay. The graph demonstrates that TFR2 silencing reduces Cu toxicity in ATP7B-KO:PRNP-KD cells (Mean ± SEM; two-way ANOVA; n=3 experiments).

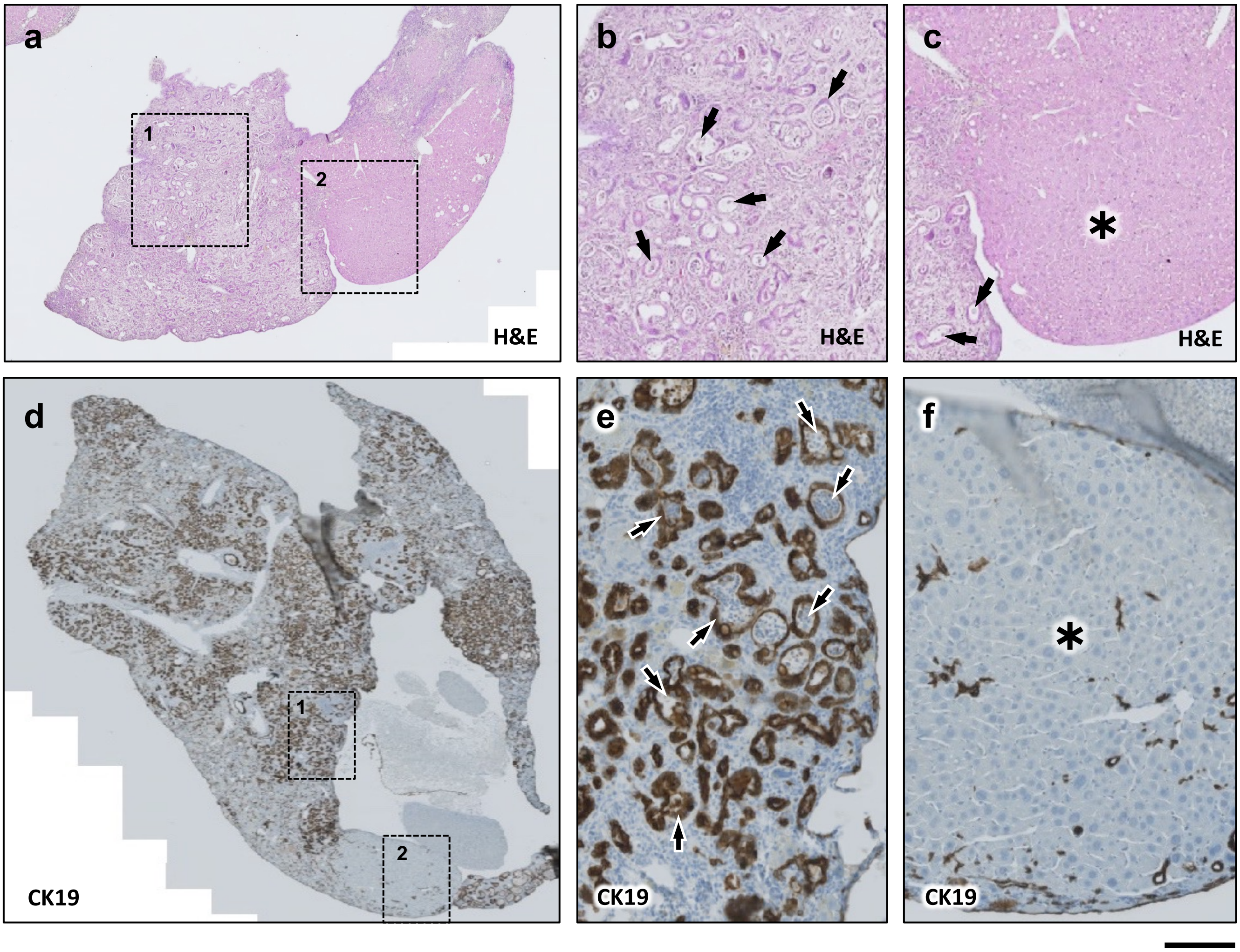

Sup. Fig. 8. Cholangiocarcinoma-like nodes in Atp7b$^{-/-}$ mice.

Liver tissue from Atp7b$^{-/-}$ mice was embedded in paraffin, sectioned and stained with hematoxylin and eosin (a-c) or with bona fide cholangiocarcinoma marker cytokeratin 19 (e-f). Entire sections were then scanned (see overall view in panels a and d). Panel b corresponds to box1 in panel a and shows cholangiocarcinoma-like node with proliferating biliary ducts (arrows). Panel c corresponds to box2 in panel a and shows regenerative node with normal morphology (asterisk) flanking the cholangiocarcinoma area with biliary ducts (arrows). Panel e corresponds to box1 in panel d and shows cholangiocarcinoma-like node with multiple CK19-positive biliary ducts (arrows). Panel f corresponds to box2 in panel d and shows regenerative node with normal morphology (asterisk) containing few small CK19-positive biliary ducts. Scale bar: 480µm (a, d), 170µm (b, c), 100 µm (e,f).

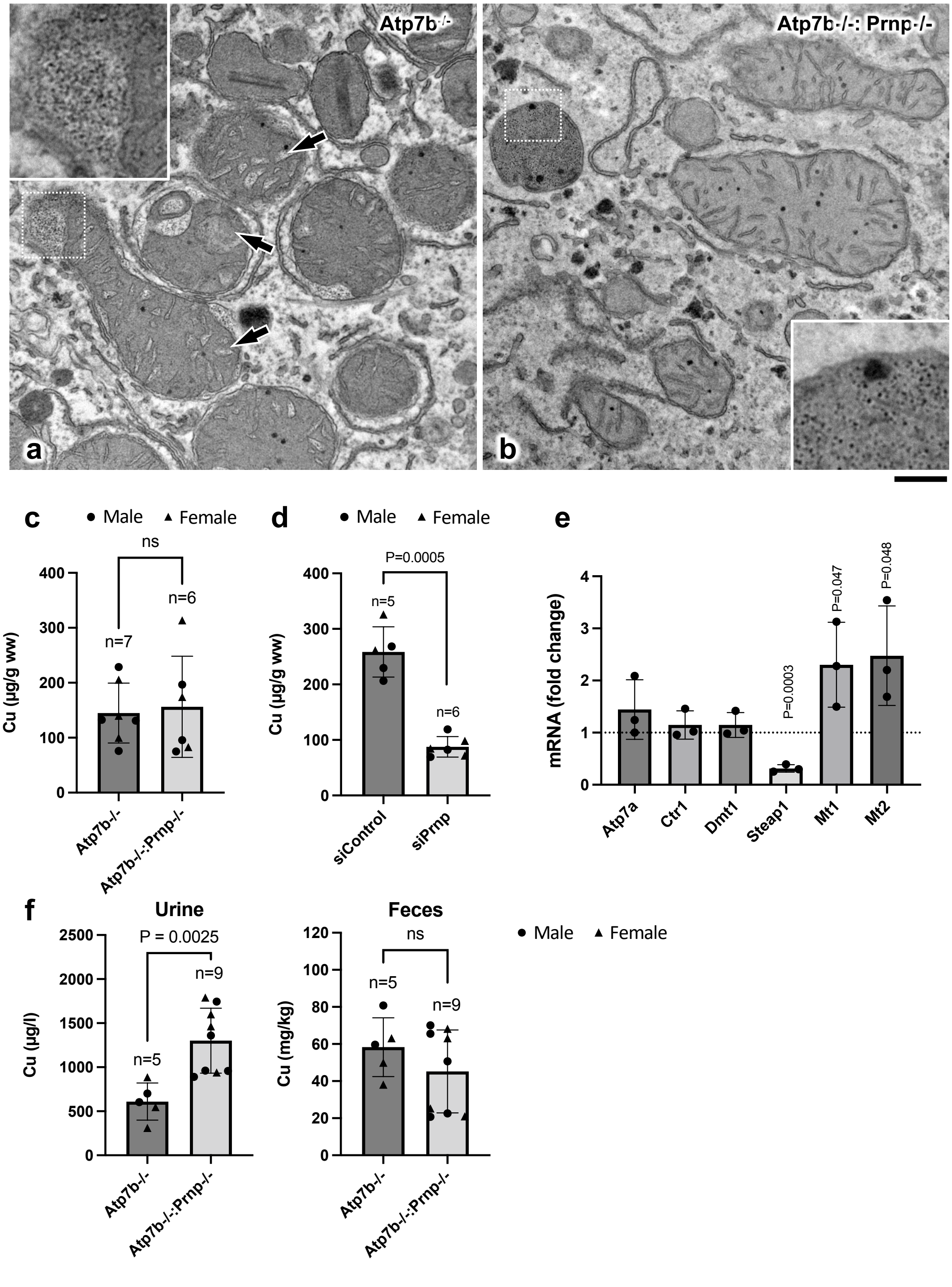

Sup. Fig. 9. Impact of *Prnp* suppression on hepatic mitochondria, copper levels and expression of copper transporters in Atp7b$^{-/-}$ mice.

a, b. EM images show mitochondria in the liver tissue of Atp7b$^{-/-}$ (a) or Atp7b$^{-/-}$: Prnp$^{-/-}$ (b) mice. Arrows and arrowheads in A indicate swollen cristae and intermembrane space respectively. Insets in a and b correspond to the dashed boxes and show accumulation of electron-dense particles in mitochondria (a) or lysosome (b). **c.** Cu concentrations were quantified in livers from Atp7b$^{-/-}$ or Atp7b$^{-/-}$: Prnp$^{-/-}$ mice using ICP-OES and expressed as µg per g of wet tissue weight (ww). No statistically significant differences were observed (Mean ± SD; t-test; n=number of animals). **d.** Atp7b$^{-/-}$ mice were injected with *Prnp*-specific siRNAs for 4 weeks (see methods) and subjected to ICP-OES analysis of hepatic Cu as described in c. The graph shows *Prnp*-specific siRNA significantly reduced Cu levels in the liver of Atp7b$^{-/-}$ animals (Mean ± SD; t-test; n=number of animals). **e.** Expression of genes encoding different Cu-transporting/binding proteins was evaluated in the liver of Atp7b$^{-/-}$ or Atp7b$^{-/-}$: Prnp$^{-/-}$ mice by QRT-PCR. The graph shows changes in mRNA levels of these genes in Atp7b$^{-/-}$: Prnp$^{-/-}$ mice compared to Atp7b$^{-/-}$ mice (dash line) (Mean ± SD; t-test; n=3 animals). **F.** The graph shows Cu levels in urine and feces measured in Atp7b$^{-/-}$ or Atp7b$^{-/-}$: Prnp$^{-/-}$ mice by ICP-OES. Atp7b$^{-/-}$: Prnp$^{-/-}$ mice exhibited higher Cu values in the urine (Mean ± SD; t-test; n=number of animals). Cu levels in feces did nor show statistically significant differences between analyzed strains. Individual values for each analyzed animal are shown in each graph (c-f). Scale bar: 260 nm (a, b).

Supplementary Table 1. Properties of validated hit genes

Gene	Metal specificity	Impact of RNAi on Cu toxicity in ATP7B-KO cells	Role in Cu metabolism	Knockout mice phenotype	Human disease due to loss of function
MMGT1	Mg	***<br>*	-	Lethal [1]	-
PRNP	Cu	****<br>**	Endocytic uptake of copper [2]	- Viable, no severe phenotype [3]<br>- Late onset peripheral neuropathy [4]	Only toxic gain [11]
SLC25A37	Fe, Cu	*<br>*	Transport of Cu to mitochondria [5]	- Whole body lethal [6]<br>- Hepatic results in reduced iron in mitochondria, but viable [6]	-
STEAP1	Fe, Cu	***<br>*	Cu reductase [7]	-	-
TFR2	Fe	*<br>****	-	Hemochromatosis-like [8]	Hemochromatosis type 3 [11]
SLC11A2	Divalent metals including Cu, Fe and Zn	*<br>*	Transport across the membrane to cytoplasm [9]	Lethal [10]	Hemochromatosis type 1 [11]

1. Brommage, R. *et al.* High-throughput screening of mouse gene knockouts identifies established and novel skeletal phenotypes. *Bone Res* **2**, (2015).
2. Perera, W. S. S. & Hooper, N. M. Ablation of the metal ion-induced endocytosis of the prion protein by disease-associated mutation of the octarepeat region. *Current Biology* **11**, (2001).
3. Bueler, H. *et al.* Normal development and behaviour of mice lacking the neuronal cell-surface PrP protein. *Nature* **356**, 577–582 (1992).
4. Bremer, J. *et al.* Axonal prion protein is required for peripheral myelin maintenance. *Nat Neurosci* **13**, 310–318 (2010).
5. Christenson, E. T., Gallegos, A. S. & Banerjee, A. In vitro reconstitution, functional dissection, and mutational analysis of metal ion transport by mitoferrin-1. *J Biol Chem* **293**, 3819–3828 (2018).
6. Seguin, A. *et al.* The mitochondrial metal transporters mitoferrin1 and mitoferrin2 are required for liver regeneration and cell proliferation in mice. *Journal of Biological Chemistry* **295**, (2020).
7. Oosterheert, W. & Gros, P. Cryo-electron microscopy structure and potential enzymatic function of human six-transmembrane epithelial antigen of the prostate 1 (STEAP1). *Journal of Biological Chemistry* **295**, (2020).
8. Wallace, D. F., Summerville, L., Lusby, P. E. & Subramaniam, V. N. First phenotypic description of transferrin receptor 2 knockout mouse, and the role of hepcidin. *Gut* **54**, (2005).
9. Arredondo, M., Muñoz, P., Mura, C. V. & Núñez, M. T. DMT1, a physiologically relevant apical Cu1+ transporter of intestinal cells. *Am J Physiol Cell Physiol* **284**, (2003).
10. Gunshin, H. *et al.* Slc11a2 is required for intestinal iron absorption and erythropoiesis but dispensable in placenta and liver. *Journal of Clinical Investigation* **115**, (2005).
11. WWW.OMIM.ORG

Uncropper Western blot for Figure3b

PrP

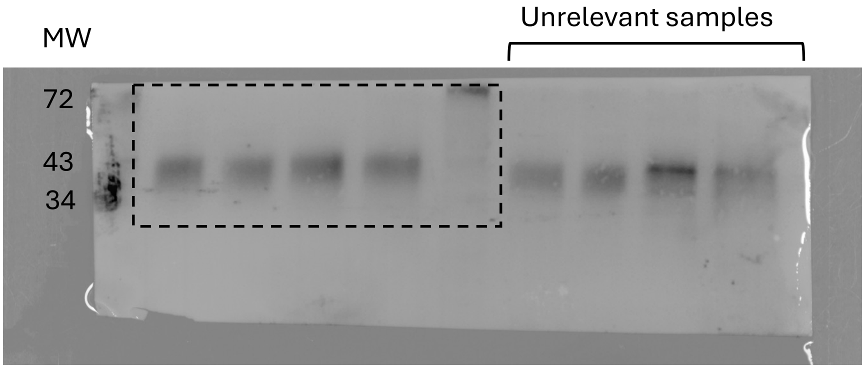

Lower exposure

PrP

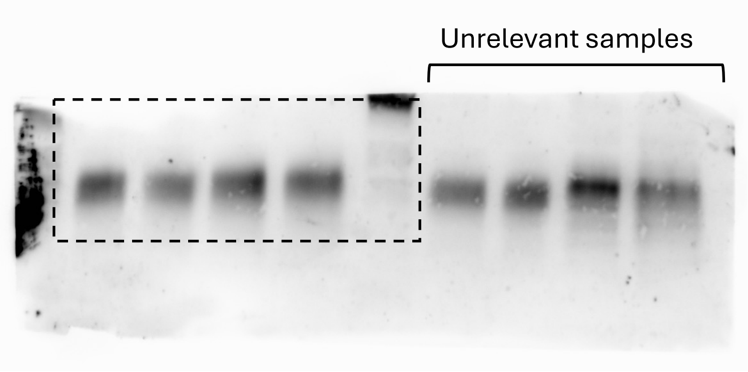

Higher exposure

Vinculin

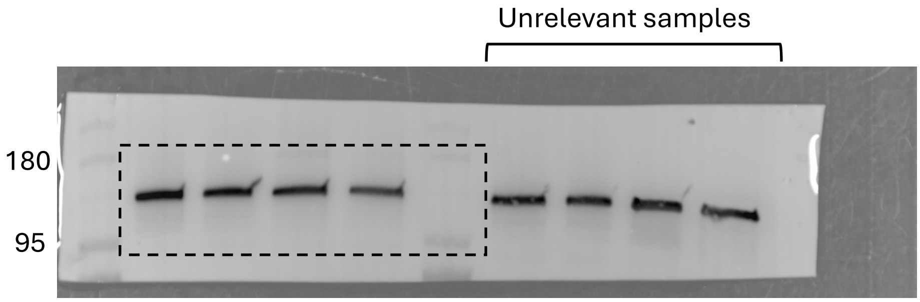

Dash rectangle indicates area included in the figure

Uncropper Western blot for Figure3e

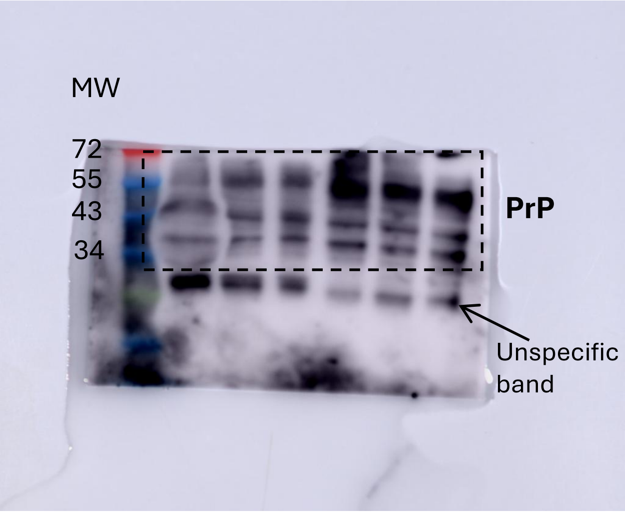

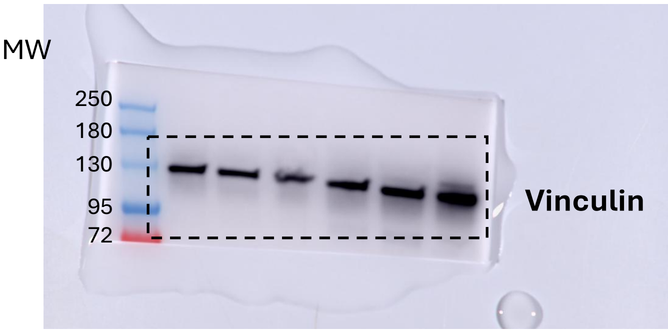

Dash rectangle indicates area included in the figure

Uncropper Western blot for Supplementary Fiure 1b

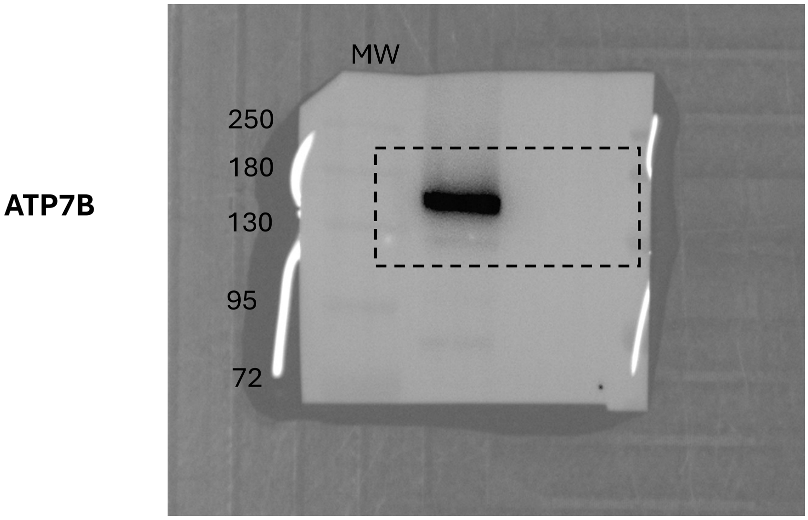

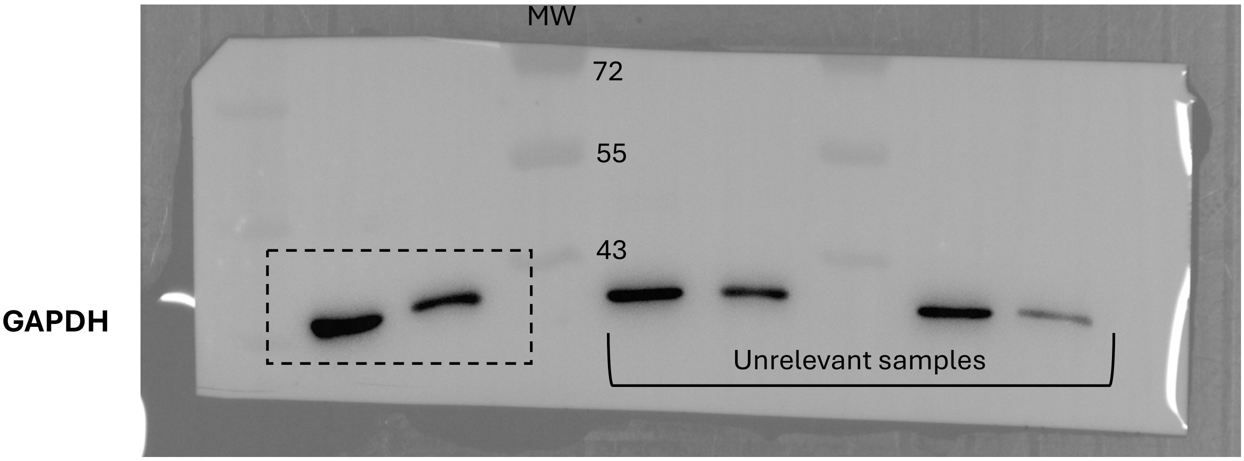

Dash rectangle indicates area included in the figure

Uncropper Western blot for Supplementary Figure 5a

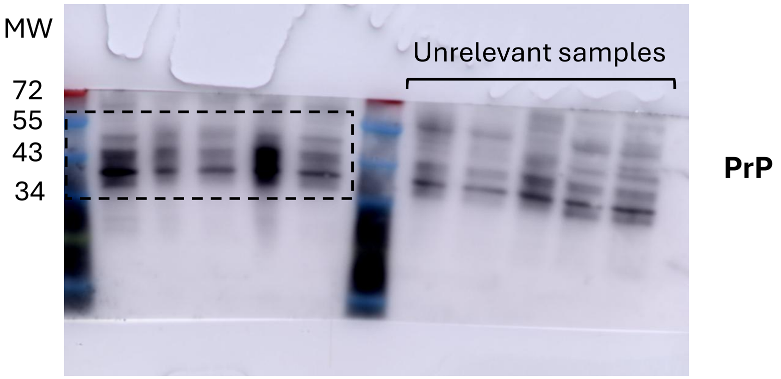

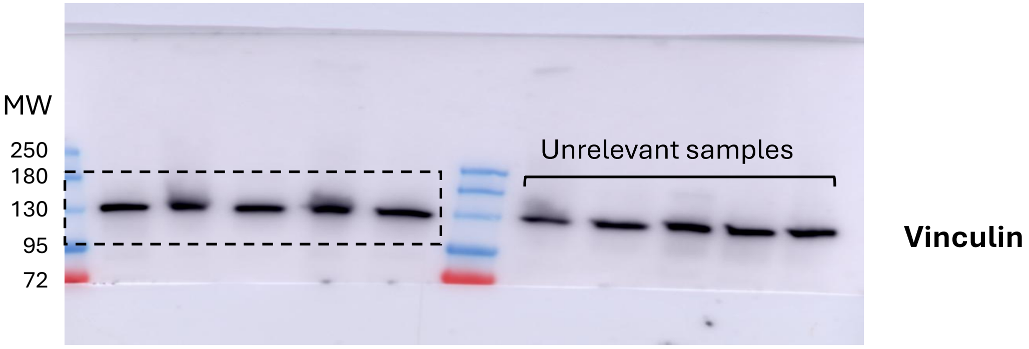

Dash rectangle indicates area included in the figure

Uncropper Western blot for Supplementary Figure 5f

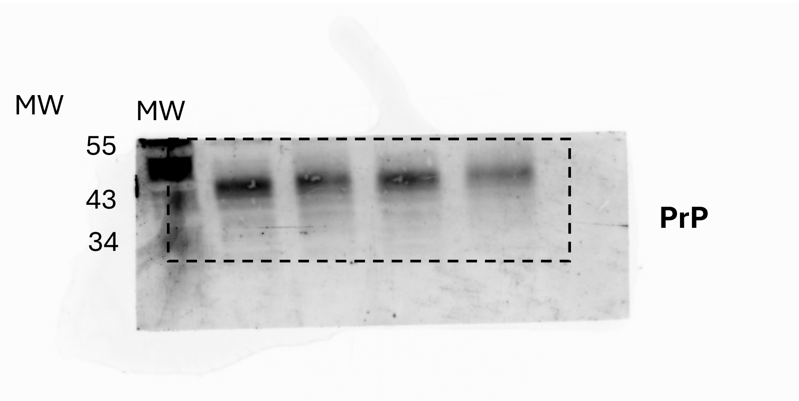

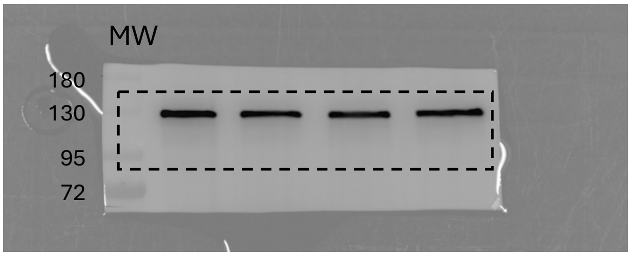

Dash rectangle indicates area included in the figure
