# Supplementary material for: Prion protein promotes copper toxicity in Wilson disease
Source: Nat Commun. 2025 Feb 8;16:1468. doi: 10.1038/s41467-025-56740-x (PMC11807206; doi:10.1038/s41467-025-56740-x)
Supplement: Supplementary file 2 — Description of Additional Supplementary Files [file 41467_2025_56740_MOESM2_ESM.pdf]

DESCRIPTION OF ADDITIONAL SUPPLEMENTARY FILES

SUPPLEMENTARY DATASET 1.
List of the shRNAs corresponding to screening hit genes, whose suppression improves resistance to copper in ATP7B-KO cells.
The gene was considered a hit if it met the following criteria (see Methods):
1) At least 2 significantly enriched (FDR<0.05) shRNAs per gene from different pools
2) At least 1 shRNA per gene with ≥2 fold enrichment (LogFC≥1)
3) No significantly depleted (FDR<0.05) shRNAs corresponding to the gene
4) Lack of corresponding hits in vinblastine screening (see supplementary table 2)

SUPPLEMENTARY DATASET 2.
List of the shRNAs corresponding to screening hit genes, whose suppression improves resistance to vinblastine in ATP7B-KO cells.
The genes, which were also detected as hits in copper toxicity screening are indicated with red text and were excluded from Supplementary dataset 1.

SUPPLEMENTARY DATASET 3.
List of the hit genes that belong to "transition metal transport/homeostasis" gene ontology (GO) categories.

SUPPLEMENTARY DATASET 4.
List of the siRNAs used in the study.

SUPPLEMENTARY DATASET 5.
List of primers used in the study.
