# Supplementary material for: Prion protein promotes copper toxicity in Wilson disease
Source: Nat Commun. 2025 Feb 8;16:1468. doi: 10.1038/s41467-025-56740-x (PMC11807206; doi:10.1038/s41467-025-56740-x)
Supplement: Supplementary file 8 — Reporting Summary [file 41467_2025_56740_MOESM8_ESM.pdf]

nature portfolio

Corresponding author(s): Roman Polishchuk and Roberto Chiesa

Last updated by author(s): Dec 18, 2024

Reporting Summary

Nature Portfolio wishes to improve the reproducibility of the work that we publish. This form provides structure for consistency and transparency in reporting. For further information on Nature Portfolio policies, see our Editorial Policies and the Editorial Policy Checklist.

Statistics

For all statistical analyses, confirm that the following items are present in the figure legend, table legend, main text, or Methods section.

n/a	Confirmed	
☐	☒	The exact sample size (*n*) for each experimental group/condition, given as a discrete number and unit of measurement
☐	☒	A statement on whether measurements were taken from distinct samples or whether the same sample was measured repeatedly
☐	☒	The statistical test(s) used AND whether they are one- or two-sided<br>*Only common tests should be described solely by name; describe more complex techniques in the Methods section.*
☐	☒	A description of all covariates tested
☐	☒	A description of any assumptions or corrections, such as tests of normality and adjustment for multiple comparisons
☐	☒	A full description of the statistical parameters including central tendency (e.g. means) or other basic estimates (e.g. regression coefficient) AND variation (e.g. standard deviation) or associated estimates of uncertainty (e.g. confidence intervals)
☐	☒	For null hypothesis testing, the test statistic (e.g. *F*, *t*, *r*) with confidence intervals, effect sizes, degrees of freedom and *P* value noted<br>*Give P values as exact values whenever suitable.*
☒	☐	For Bayesian analysis, information on the choice of priors and Markov chain Monte Carlo settings
☒	☐	For hierarchical and complex designs, identification of the appropriate level for tests and full reporting of outcomes
☒	☐	Estimates of effect sizes (e.g. Cohen's *d*, Pearson's *r*), indicating how they were calculated

Our web collection on statistics for biologists contains articles on many of the points above.

Software and code

Policy information about availability of computer code

Data collection	Fluorescence micrographs were collected using Zeiss LSM800 or LSM710 confocal system (Zeiss, Germany) equipped with an Electronically Switchable Illumination and Detection (ESID) module and controlled by Zen blue software for LSM800 (v.2.6) and Zen 2012 for LSM 710. EM images were acquired using a FEI Tecnai-12 electron microscope (FEI, Eindhoven, Netherlands) equipped with a VELETTAC CDd igital camera (Soft Imaging SystemsG mbH, Munster, Germany). Morphometric analysis was performed using iTEM software (Olympus SYS, Germany, v.5.2).
Data analysis	Fluorescence images were processed with Fiji (ImageJ v.1.51j8). Morphometric analysis of EM images was performed using iTEM software (Olympus SYS, Germany, v.5.2). Details of NGS data analysis are provided in Materials and Methods. To measure the relative quantity of each shRNA in a sample, we aligned the NGS reads to the construct FASTA reference files, provided with the Dharmacon library, using Bowtie2 (ver. 2.4.2) and Python (ver. 2.7.18). Normalized raw counts for shRENAs and their statistical analyses were counducted in R environment (ver. 4.2.3), using NOISeq package (ver. 2.42.0) for the normalization 52,53, and edgeR package (ver. 3.40.2). Statistical analyses of non-NGS data were performed using GraphPad Prism (Graph Pad Software Inc v.9 or 10).

For manuscripts utilizing custom algorithms or software that are central to the research but not yet described in published literature, software must be made available to editors and reviewers. We strongly encourage code deposition in a community repository (e.g. GitHub). See the Nature Portfolio guidelines for submitting code & software for further information.

Data

Policy information about availability of data

All manuscripts must include a data availability statement. This statement should provide the following information, where applicable:

- Accession codes, unique identifiers, or web links for publicly available datasets
- A description of any restrictions on data availability
- For clinical datasets or third party data, please ensure that the statement adheres to our policy

Sequencing data were deposited to Gene Expression Omnibus (GEO repository) with following accession numbers:
GSE277865 (https://www.ncbi.nlm.nih.gov/geo/query/acc.cgi?acc=GSE277865),
GSE260454 (https://www.ncbi.nlm.nih.gov/geo/query/acc.cgi?acc=GSE260454).
Results of NGS and Gene Ontology Enrichment analyses are available in the supplementary datasets. Light and electron microscopy images supporting the microscopy data shown in the figures have been deposited in a publicly accessible Zenodo repository and are available at the following links: https://doi.org/10.5281/zenodo.14500237 and https://noprofittigem.sharepoint.com/sites/PolishchukLabPapers.
Full uncropped images of Western blots are available in the supplementary information file. All statistical data presented in the graphs are included in the Source Data file.

Research involving human participants, their data, or biological material

Policy information about studies with human participants or human data. See also policy information about sex, gender (identity/presentation), and sexual orientation and race, ethnicity and racism.

Reporting on sex and gender	N/A
Reporting on race, ethnicity, or other socially relevant groupings	N/A
Population characteristics	N/A
Recruitment	N/A
Ethics oversight	N/A

Note that full information on the approval of the study protocol must also be provided in the manuscript.

Field-specific reporting

Please select the one below that is the best fit for your research. If you are not sure, read the appropriate sections before making your selection.

☒ Life sciences ☐ Behavioural & social sciences ☐ Ecological, evolutionary & environmental sciences

For a reference copy of the document with all sections, see nature.com/documents/nr-reporting-summary-flat.pdf

Life sciences study design

All studies must disclose on these points even when the disclosure is negative.

Sample size	For in vivo experiments power analysis (using GPower) was performed to determine the sufficient number of animals to be assigned to each experimental group. For in vivo experiments the no sample size calculation was done. Experiments were repeated at least three times with similar results and sample size was chosen based on the consistency and significance of measured differences between groups and or conditions.
Data exclusions	No data exclusions.
Replication	Each experiment in the manuscript was repeated at least three times (unless otherwise stated) under standard and clearly defined conditions; all attempts at replication were successful.
Randomization	Samples are randomly assigned to experimental groups, to processing order, or to positions in a multi-well device. Images were selected randomly and analyzed equally, no sub-sampling so no randomization was necessary.
Blinding	Blinding was not relevant for in vivo experiments. For in vitro experiments the specimens were blinded for microscopy analyses.

Reporting for specific materials, systems and methods

We require information from authors about some types of materials, experimental systems and methods used in many studies. Here, indicate whether each material, system or method listed is relevant to your study. If you are not sure if a list item applies to your research, read the appropriate section before selecting a response.

Materials & experimental systems

n/a	Involved in the study
☐	☒ Antibodies
☐	☒ Eukaryotic cell lines
☒	☐ Palaeontology and archaeology
☐	☒ Animals and other organisms
☒	☐ Clinical data
☒	☐ Dual use research of concern
☒	☐ Plants

Methods

n/a	Involved in the study
☒	☐ ChIP-seq
☒	☐ Flow cytometry
☒	☐ MRI-based neuroimaging

Antibodies

Antibodies used	The followed antibodies were used: mouse monoclonal anti-PrP 3F4 (Chiesa, Tapella et al. Biochem.J. (2013)454,417–425, dilution:1:100 for IF), mouse monoclonal anti-PrP 6D12 (Chiesa, Tapella et al. Biochem.J. (2013)454,417–425, dilution: 1:100 for IF), mouse monoclonal anti-PrP 12B2, 100B3, 94B4 (Wageningen University and Research, dilution: 1:100 for IF, 1:1000 for WB, 1:100 for IHC); mouse monoclonal anti-MRP2 (Enzo Lifescience, ALX-801-016-C250,dilution: 1:50 for IF); mouse monoclonal anti-PrP SAF32 (Bertin Bioreagent, A03202, dilution: 1: 50 for IF), rabbit anti-Myc tag (Millipore, 06549, dilution 1:100), mouse monoclonal anti-K/ Na-ATPase (Abcam, ab7671,dilution 1:100), mouse anti-GAPDH (Santa Cruz Biotechnology, sc-32233, dilution 1:1000 for WB), rabbit monoclonal anti-ATP7B (Abcam, Ab 124973, dilution 1:1000 for WB) or mouse monoclonal anti-Vinculin (Sigma-Aldrich-Aldrich, V9264, dilution 1:5000 for WB). Secondary antibodies for IF were Alexa Fluor-488 (A11008, A11001)-568 (A11011, A11004)-647 (A31573) (Invitrogen, diluted 1:400). Secondary antibodies for WD were horseradish peroxidase (HRP)-conjugated goat anti-mouse or anti-rabbit IgG antibody (1:8,000, Merck Millipore, 401215 and 401315, respectively).
Validation	Most of the antibodies used in the study were bought from commercial vendors and were validated by the manufacturers and/ or other studies (info is available at the product-corresponding web pages). Reference for non commercial antibodies is provided.

Eukaryotic cell lines

Policy information about cell lines and Sex and Gender in Research

Cell line source(s)	Cell line sources: HepG2 cells were obtained from ATCC. HepG2 cells with ATP7B knockout were a kind gift from Andree Zibert (Universitätsklinikum Münster, Münster, Germany), originally published in Chandhok et al. (PLOS One, 2014). HepG2 cells with ATP7B knockout and stable PRNP shRNA-mediated knockdown were generated in this study.
Authentication	All stable cell lines were authenticated by QRT-PCR, WB or IF. Commercial cell lines were purchased recently from ATCC and validated by morphological analysis.
Mycoplasma contamination	Mycoplasma contamination: Cell lines were routinely tested negative for mycoplasma by RT-PCR
Commonly misidentified lines (See ICLAC register)	No commonly misidentified lines were used.

Animals and other research organisms

Policy information about studies involving animals; ARRIVE guidelines recommended for reporting animal research, and Sex and Gender in Research

Laboratory animals	Atp7b-/- and PrP-/- mouse strains were used in the study and maintained on C57BL/6 × 129S6/SvEv and C57BL/6 background, respectively. The age of animals was from 12 to 28 weeks.
Wild animals	N/A
Reporting on sex	Both males and females were enrolled in the study, following stringent ethical requirements mandated by governmental authorities to minimize animal use. Each experimental group included a similar number of males and females, and no specific sex-related studies were conducted.
Field-collected samples	N/A
Ethics oversight	Protocols for animal use were approved by Italian Government (Ministry of the Health) and by ethical committee of Research

Ethics oversight | Institute of Experimental Medicine, St. Petersburg, (Russia).

Note that full information on the approval of the study protocol must also be provided in the manuscript.

Plants

Seed stocks | N/A

Novel plant genotypes | N/A

Authentication | N/A
